# O042. Phase-dependent defective functional activity of the default mode network and facilitated temporal processing of nociceptive stimuli in cluster headache

**DOI:** 10.1186/1129-2377-16-S1-A90

**Published:** 2015-09-28

**Authors:** Armando Perrotta, Maria Grazia Anastasio, Luigi Pavone, Antonio Ferretti, Piero Chiacchiaretta, Giovanni Grillea, Marcello Bartolo, Emanuele Siravo, Gianluca Coppola, Anna Ambrosini, Roberto De Icco, Giorgio Sandrini, Francesco Pierelli

**Affiliations:** 1 IRCCS INM Neuromed, Pozzilli, Italy; 2 Department of Medical and Surgical Sciences and Biotechnologies, “Sapienza” University of Rome Polo Pontino, Rome, Italy; 3 Department of Neuroscience, Imaging and Clinical Sciences, University “G. d'Annunzio”, Institute for Advanced Biomedical Technologies (ITAB), Chieti, Italy; 4 IRCCS Neurological National Institute C. Mondino, Pavia, Italy

## Background

In cluster headache (CH) during the active period we described a facilitated temporal summation (TS) of nociceptive signals at spinal level linked to a defective suprapinal control of pain and followed by a normalization of the values during the remission period[1]. TS of sensory neuronal responses to nociceptive stimuli is a form of central plasticity that shifts the sensory information from tactile to nociceptive before transmitting the nociceptive information to brain areas mediating pain sensation. This feature of the sensory system results pivotal in physiological nociception, for discrimination between innocuous and potentially dangerous stimulation, as well as in pathological nociception, for induction and maintenance of the central sensitization, subsequently resulting in pain chronification[2]. In this study we sought to determine which brain sites are involved in the modulation of temporal processing of pain sensation in CH subjects during both the active and remission period. We utilized functional magnetic resonance imaging (fMRI) to compare the Blood Oxygenation Level Dependent (BOLD) signal changes related to the temporal summation threshold (TST) of the nociceptive withdrawal reflex (NWR). We used the single NWR response as control stimulus.

## Methods

We studied 10 episodic CH patients during both active and remission period and 17 healthy subjects (HS). Two types of stimulation blocks were delivered during the fMRI scanning according to the stimulation paradigms previously determined to evoke both the TST of the NWR (SUMM) and the NWR single response (SING).

## Results

The analysis of the hemodynamic signals showed a comparable activation of sensory and pain related areas in both CH (during active and remission period) and HS. The most relevant differences emerged in the deactivation of both posterior cingulate cortex (PCC) and bilateral angular gyrus (AG) and in the activation of the anterior cingulate cortex (ACC). CH during the active phase showed a lack of deactivation of PCC and AG and a more relevant activation of the ACC when compared to CH during the remission phase and HS.

## Conclusions

PCC, AG and ACC are considered to be pivotal in default mode network (DMN), with a high activity correlated to the rest and reactive deactivation during most tasks where the attention is directed externally. Our data have demonstrated that in CH during the active phase of the disease, the facilitation in temporal processing of nociceptive stimuli is linked to a defective functioning of the DMN. Interestingly, both these abnormalities are dependent on the clinical activity of the disease.

Written informed consent to publication was obtained from the patient(s).
